# Cross-Species Analysis of Transcriptomic Response to Alpha-Herpesvirus Infection in Human, Bovine and Equine Cells

**DOI:** 10.3390/ijms27031261

**Published:** 2026-01-27

**Authors:** Mirko Schmitz, Eva Neugebauer, Florian Full, Kristen L. Conn

**Affiliations:** 1 Institute of Virology, Medical Center, University of Freiburg, 79104 Freiburg, Germany; 2 Spemann Graduate School of Biology and Medicine (SGBM), University of Freiburg, 79104 Freiburg, Germany; 3 Faculty of Biology, University of Freiburg, 79104 Freiburg, Germany; 4 German Consulting Laboratory for HSV and VZV, Medical Center, University of Freiburg, 79104 Freiburg, Germany; 5 Department of Veterinary Microbiology, Western College of Veterinary Medicine, University of Saskatchewan, Saskatoon, SK S7N 5B4, Canada

**Keywords:** herpesvirus, alphaherpesvirus, HSV-1, BHV-1, bovine, EHV-1, equine, RNA sequencing, transcriptomics, orthologous gene mapping

## Abstract

Comparative transcriptomics offers a powerful approach to elucidate host–virus interactions across related pathogens, yet systematic evaluations across species-matched cellular systems remain limited. We performed a cross-species RNA sequencing analysis of respective species’ cells infected with three alphaherpesviruses—herpes simplex virus 1 (HSV-1), bovine alphaherpesvirus 1 (BHV-1), and equid alphaherpesvirus 1 (EHV-1)—to dissect conserved and virus-specific transcriptional responses. We show that certain orthologous genes and orthologous pathways are differentially regulated upon infection among the three species like pathways related to translation rRNA processing and TNF-alpha signalling. We find that the earliest sampled timepoint of infection, 2 h post infection (hpi), shows the most commonly enriched pathways among the three species compared to later timepoints. At 6 h and 9 h post infection, BHV-1- and EHV-1 infections have more in common with each other in terms of enriched pathways than with HSV-1 infections. Moreover, we provide a comprehensive analysis of temporal viral gene expression for all three herpesviruses. Together, these findings provide a comparative framework for understanding alphaherpevirus–host interactions and reveal both conserved core responses and species-specific transcriptional signatures. This work establishes a foundation for identifying broadly acting antiviral targets as well as virus-specific vulnerabilities that may inform host-directed therapies and cross-species disease management.

## 1. Introduction

Alphaherpesviruses are enveloped double-stranded DNA viruses with a high genetic population diversity and a wide species range [1]. In contrast to other virus families that can have broad host ranges, herpesviruses are predominantly species-specific and share millions of years of coevolution with their respective hosts [2]. Bovine alphaherpesvirus 1 (BHV-1) is a major pathogen of cattle and a member of the genus *Varicellovirus* within the family Herpesviridae [3]. BHV-1 is responsible for a wide range of clinical syndromes collectively referred to as infectious bovine rhinotracheitis (IBR), including respiratory disease, conjunctivitis, vulvovaginitis, balanoposthitis, abortion, and enteritis [4,5]. The virus has a global distribution and causes considerable economic losses in the cattle industry as a result of decreased productivity, reproductive failure, and trade restrictions [6,7]. In addition, BHV-1 is a key component of the bovine respiratory disease complex (BRDC), commonly known as “shipping fever,” predisposing infected animals to secondary bacterial infections [8].

Transmission of BHV-1 occurs through various routes, including aerosol inhalation, direct sexual contact, contaminated semen used for artificial insemination, and vertical transmission across the placenta [9,10]. The clinical expression of disease depends largely on viral strain virulence, host immune status, and environmental stress factors [11]. Although BHV-1 infections are seldom fatal, they can significantly impair herd health and productivity [11,12]. Following acute infection, BHV-1 establishes lifelong latency in sensory neurons, predominantly within the trigeminal and sciatic ganglia [13]. Periodic reactivation and viral shedding may occur under stress or immunosuppressive conditions, enabling persistent circulation of the virus within cattle populations [14,15]. As with other alphaherpesviruses, the ability of BHV-1 to alternate between lytic and latent phases facilitates its epidemiological persistence and complicates eradication programmes [13].

Equid alphaherpesvirus 1 (EHV-1) is a highly significant pathogen of horses and, like BHV-1, belongs to the genus *Varicellovirus* within the family Herpesviridae [3]. EHV-1 is associated with diverse clinical manifestations, including respiratory disease, abortion, neonatal foal death, and equine herpesvirus myeloencephalopathy (EHM), a severe neurological syndrome [16,17]. The virus is endemic in equine populations worldwide and poses a major threat to the global horse industry due to its effects on animal welfare, performance, and trade [17,18]. Transmission occurs primarily via aerosolized respiratory secretions, direct or indirect contact with infected horses, and exposure to contaminated fomites; vertical transmission through the placenta can also lead to abortion [19]. Disease severity depends on host immunity, viral strain characteristics, and environmental conditions. While respiratory disease is typically mild and self-limiting, abortion storms and neurologic outbreaks can cause significant morbidity, mortality, and economic loss [17,18].

After primary infection, EHV-1 establishes lifelong latency within sensory neurons and lymphoid tissues, particularly in the trigeminal ganglia [20]. Viral reactivation, often triggered by stress or immunosuppression, leads to renewed shedding and facilitates maintenance of infection within equine populations [21]. Similarly to BHV-1, the capacity of EHV-1 to alternate between latent and lytic phases underlies its persistence and the difficulty of achieving effective long-term control [22,23].

Comparative transcriptomic studies provide a powerful framework for elucidating both conserved and host-specific mechanisms underlying virus biology. BHV-1 and EHV-1 share a close phylogenetic relationship within alphaherpesviruses, yet they exhibit distinct host ranges, tissue tropisms, and disease manifestations [24]. Transcriptomic profiling enables comprehensive identification of viral gene expression dynamics across different stages of infection—lytic replication, latency establishment, and reactivation—and allows for the characterization of host cellular responses specific to each virus–host interaction [25,26]. By comparing global gene expression patterns across these viruses, it becomes possible to identify conserved regulatory networks, promoter motifs, and latency-associated transcripts that are critical for viral persistence [27]. Moreover, cross-species analyses may reveal divergent transcriptional strategies that explain host adaptation, immune evasion, and neuropathogenesis. Such comparative data not only enhance our understanding of the molecular evolution of alphaherpesviruses but also provide valuable insights into potential therapeutic and vaccine targets applicable across multiple species, as has been shown for other viruses [28,29].

Several important publications from the Boldogkoi lab investigated the transcriptome of EHV-1- and BHV-1-infected cells, mostly using long-read sequencing [30,31,32,33]. Their work enabled the analysis of temporal viral gene expression, annotation of transcriptional start sites (TSSs), annotation of transcriptional end sites, as well as the identification of viral transcript and splicing variants. However, the studies performed by Tombacz et al. that focused on EHV-1 were performed in RK-13 cells, a rabbit cell line that is commonly used for propagation and infection studies of EHV-1 [31,33]. Such species-mismatched infection does not provide any insight into the cellular transcriptomic responses or infection progression within cells of the natural host species. In addition, Jiang et al. investigated the effect of IFN-gamma treatment on the transcriptome of BHV-1-infected MDBK cells and demonstrated that IFN-gamma increases expression of host immune genes, however the authors do not show expression data for herpesviral transcripts [25]. Therefore, comparative analyses of cellular host responses to alpha-herpesviral infections are missing that could highlight evolutionarily conserved pathways that are up- or downregulated in response to infection, including those that are pro- or antiviral.

In this study, we aimed to analyze the cellular transcriptomes of BHV-1 or EHV-1-infected species-matched cells and compare their temporal transcriptomic profiles with the profile of human cells infected with HSV-1. This direct cross-species comparison identified orthologous genes that are differentially expressed as well as pathways that are either up- or downregulated among all three viral infection-mediated host responses, indicating a conserved cellular response to viral infection in different species.

## 2. Results

**Figure 1:**
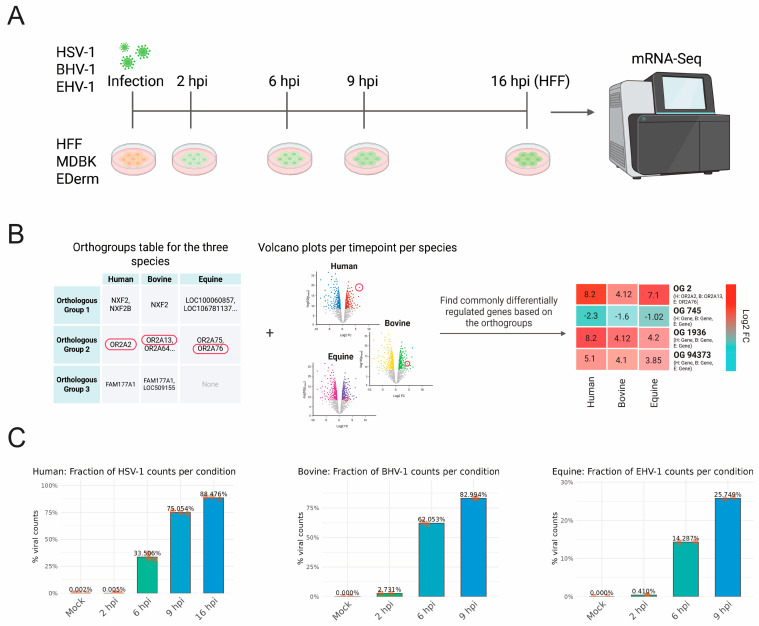
Overview of experimental design and data processing procedure. (**A**) Illustration depicting the different species’ cells, the corresponding species’ alphaherpesvirus, and the timepoints samples in the RNA sequencing experiment. For all samples, the following timepoints were sampled: mock, 2 hpi, 6 hpi, and 9 hpi. For the HFF cells, 16 hpi was also sampled. (**B**) Illustration showing how orthologous gene groups were used to infer differentially expressed orthologous genes of multiple species upon infection. The OrthoFinder v3.1 tool was used to generate orthologous groups between all three species based on the proteins’ sequences, exemplary genes are encircled in red. Next, it was determined whether there were any gene groups where at least one gene per species was significantly differentially expressed. If this was the case, then the genes belonging to the same orthologous group were plotted in a heatmap. If multiple genes of one species in the same orthogroup were significantly differentially expressed then the gene with the highest absolute log2 fold change was used for the heatmap. (**C**) Fractions of viral counts for human, bovine, and equine samples in relation to total reads depicted per timepoint sampled. The mean value per condition is shown in the barplot with the replicates’ percentages represented by the dots.

The objective of this study was to use RNA sequencing to identify conserved human, bovine and equine cellular transcriptomic responses to species-matched alphaherpesvirus infections. It is hoped that the identification of cellular responses that are conserved across evolutionarily distinct species will highlight novel avenues for the development of new therapeutic strategies with efficacy in diverse species. In this study, we generated RNA sequencing data from three distinct cell lines: human foreskin fibroblast (HFF) cells infected with HSV-1, bovine kidney epithelial (MDBK) cells infected with BHV-1, and equine dermal fibroblast (EDerm) cells infected with EHV-1. The experimental design involved infection of the cells with their respective alphaherpesvirus, and analysis of infected cells at multiple times after infection (2, 6, 9, and, exclusively for the human samples, 16 h post infection; hpi) to support temporal analysis of cellular transcriptomic responses as infection progressed (Figure 1A). For all analyses of differentially expressed genes, an adjusted *p*-value (*p*adj) threshold of 0.05 was used, combined with an absolute log2 fold change threshold of 1, unless otherwise noted. A significant challenge in the realm of comparative transcriptomics across diverse species pertains to the annotation of genes, a process that is often incomplete in the case of veterinary animal species. This limitation is further compounded by a paucity of knowledge concerning gene regulation in veterinary animals. To address this challenge, human, bovine, and equine genes were clustered into orthologous groups based on their respective protein sequences using the OrthoFinder (v3.1) tool [34,35] (Table S1). This analysis enabled the identification of genes that are commonly up- or downregulated during viral infection (Figure 1B). This approach facilitated the formulation of hypotheses concerning commonly regulated genes, even in the absence of gene annotations. Furthermore, a table for HSV-1, BHV-1 and EHV-1 orthologous genes was generated for comparative purposes (Table S1). The percentage of viral reads was greater for BHV-1 across all evaluated times, and lower for EHV-1 at 6 and 9 hpi (Figure 1C). Notably, at 6 hpi, the percent of BHV-1 reads (62%) was approximately double that of HSV-1 (34%), which in turn was approximately double that of EHV-1 (14%) (Figure 1C).

**Figure 2:**
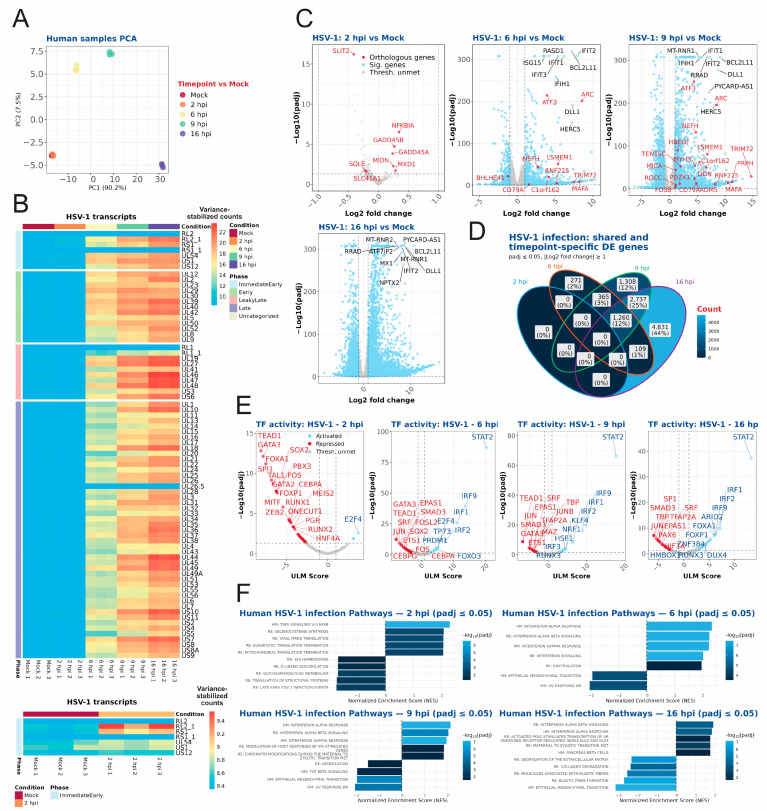
RNA sequencing analysis of human HFF cells infected with HSV-1. (**A**) Principal component analysis shown for the different timepoints and replicates of the human samples, with the variance shown for the principal components. (**B**) HSV-1 transcripts for each timepoint and replicate. The transcripts are ordered according to their respective phase in viral infection. At the bottom, the immediate early genes comparing mock samples and 2 hpi samples are shown. (**C**) Volcano plots shown for the timepoints 2 hpi, 6 hpi, 9 hpi, and 16 hpi, with significantly (*p*adj ≤ 0.05) differentially expressed genes (absolute log2 fold change of >1) shown in blue. Genes that have significantly regulated orthologous genes in all three species are depicted in red and genes below the thresholds are shown in grey. (**D**) Venn diagram showing significantly regulated (absolute log2 fold change of ≥1 and *p*adj ≤ 0.05) genes that overlap between the different timepoints of infection. (**E**) Transcription factor activity inference plots for 2 hpi, 6 hpi, 9 hpi and 16 hpi with Univariate Linear Model (ULM) score plotted with the *p*adj value. Activated transcription factors are shown in blue (ULM score ≥ 1 and *p*adj ≤ 0.05) and repressed transcription factors are shown in red (ULM score ≤ −1 and *p*adj ≤ 0.05). Transcription factors below the thresholds are shown in grey. (**F**) The most enriched significant (*p*adj ≤ 0.05) pathways for each infection timepoint (2 hpi, 6 hpi, 9 hpi, and 16 hpi) shown. For each timepoint, a maximum of 5 positively enriched and a maximum of 5 negatively enriched pathways, each with the largest absolute normalized enrichment score, are shown. All significant pathways are shown in Figure S1.

Principal component analysis (PCA) of the processed sequencing files for human cells infected with HSV-1 demonstrated distinct clustering of the replicates at each timepoint. Specifically, the 2 hpi and mock (negative control) samples exhibited close proximity in the PCA plot, while the 6, 9, and 16 hpi samples were distributed in a more dispersed manner (Figure 2A). The analysis of viral transcripts revealed the expression of immediate early genes *RS1* and *RL2* at all four timepoints (in the viral gene heatmap, there are two *RS1*, *RL1*, and *RL2* genes, since they are also present in the terminal repeats. To attribute the reads to only one copy of each gene, the terminal repeats were masked in the viral genome file, which is why *RS1_1*, *RL1* and *RL2* have counts of zero) with 2 hpi showing strong expression of these genes in comparison to the other genes at that timepoint (see Figure 2B, upper and bottom panel). At 6 hpi, early and intermediate genes are predominantly expressed, with late genes also commencing expression. Conversely, at 9 hpi and 16 hpi, late genes appear to be expressed, along with all viral genes (Figure 2B, upper panel).

As infection progressed, the number of host genes that were significantly differentially expressed increased considerably (Figure 2C, Table S2). There were around 12% overlapping differentially regulated and significant genes between 6, 9, and 16 hpi, but also numerous that were exclusive to a specific timepoint (2% for 6 hpi, 12% for 9 hpi, and 44% for 16 hpi) (Figure 2D). Based upon the differentially regulated genes, we performed transcription factor activity inference based upon the DoRothEA gene regulatory network (Figure 2E, Table S3) [36,37]. In particular, this analysis showed activation of several interferon regulatory factors (*IRF1*, *IRF2*, *IRF9*, and *IRF3*) in the later timepoints (6 hpi, 9 hpi and 16 hpi).

Pathway analysis, based upon gene set enrichment analysis (gsea) approach, was conducted utilizing a combination of the Hallmark and Reactome gene sets (Figure 2F and Figure S1). This analysis revealed the enrichment of several pathways at 2 hpi, including the TNFA signalling via NFKB, eukaryotic translation termination, and viral mRNA translation pathways. At 6 and 9 hpi, differentially upregulated genes were enriched in additional pathways, including interferon signalling, chromatin modification, DNA methylation, regulation of retroelements, and maternal-to-zygotic transition (MZT), among others (Figure S1, Table S4). The pathway analysis was also carried out through an over-representation analysis (ORA) approach, allowing us to compare the overlap between both approaches (Figure S2A,B, Table S5). ORA does show overlap with gsea but also has some exclusive pathways that are not found in gsea. We opted for gsea as the main approach because it utilizes a full ranked gene list instead of customizable thresholds for significant genes, and therefore may catch more subtle changes than ORA.

**Figure 3:**
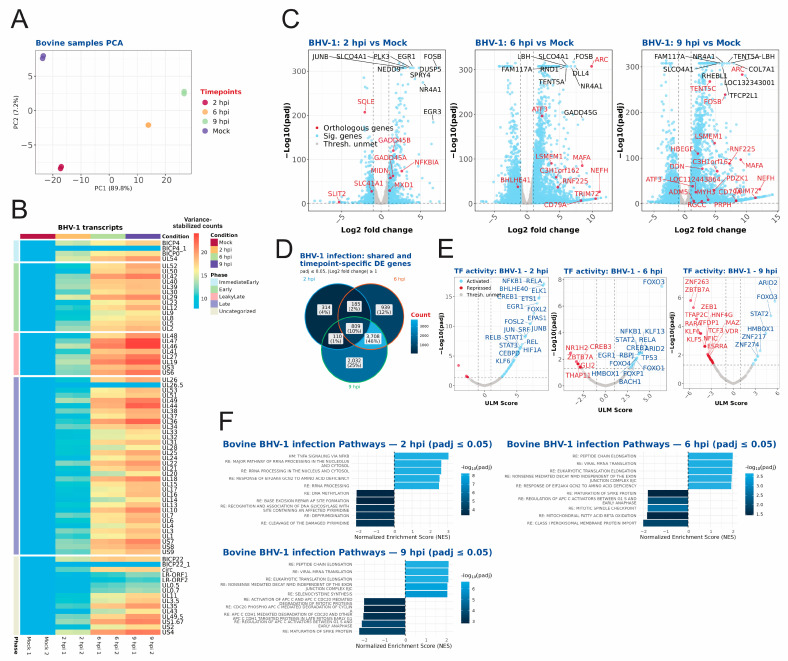
RNA sequencing analysis of bovine MDBK cells infected with BHV-1. (**A**) Principal component analysis shown for the different timepoints and replicates of the bovine samples, with the variance shown for the principal components. (**B**) BHV-1 transcripts for each timepoint and replicate. The transcripts are ordered according to their respective phase in viral infection; the phase classification is based upon the orthologous genes in human HSV-1 and their respective phase classification. (**C**) Volcano plots shown for the 2 hpi, 6 hpi, and 9 hpi timepoints, with significantly (*p*adj ≤ 0.05) differentially expressed genes (absolute log2 fold change of >1) shown in blue. Genes that have significantly regulated orthologous genes in all three species are depicted in red and genes below the thresholds are shown in grey. (**D**) Venn diagram showing significantly regulated (absolute log2 fold change of ≥1 and *p*adj ≤ 0.05) genes that overlap between the different timepoints of infection. (**E**) Transcription factor activity inference plots for 2 hpi, 6 hpi, and 9 hpi with the Univariate Linear Model (ULM) score plotted with the *p*adj value. Activated transcription factors are shown in blue (ULM score ≥ 1 and *p*adj ≤ 0.05) and repressed transcription factors are shown in red (ULM score ≤ −1 and *p*adj ≤ 0.05). Transcription factors below the thresholds are shown in grey. (**F**) The most enriched significant (*p*adj ≤ 0.05) pathways for each infection timepoint (2 hpi, 6 hpi, and 9 hpi) are shown. For each timepoint, a maximum of 5 positively enriched and a maximum of 5 negatively enriched pathways, each with the largest absolute normalized enrichment score, are shown. All significant pathways are shown in Figure S3.

In BHV-1-infected bovine cells, the replicates for each timepoint demonstrated a high degree of cohesion in the PCA plot, with each timepoint post infection exhibiting clear separation (Figure 3A). BHV-1 gene expression at 2 hpi already included expression of numerous genes, in contrast to the limited number of HSV-1 genes expressed at the same time. The differences in kinetics of BHV-1 and HSV-1 gene expression may be attributable to the higher multiplicity of infection (MOI) utilized for BHV-1 (MOI 10 for BHV-1 vs. MOI 3 for HSV-1) or alternatively, to differences in HFF and MDBK cellular permissiveness to infection (Figure 3B). As expected, BHV-1 gene expression increased over time, with higher expression levels observed for genes classified as leaky-late or late through the orthologous groups between human and bovine samples. There is no complete classification of BHV-1 genes into kinetic classes (immediate–early, early, intermediate or leaky-late and late); therefore, we tried to group BHV-1 genes into respective kinetic classes based on existing data from orthologous genes of HSV-1 (Figure 3B). Although this classification is not ideal because it neglects experimental results about temporal expression of single viral genes, it gives a complete overview of viral gene expression kinetics across the entire viral genome and shows how the non-human species’ orthologous genes’ expression profiles compare to the genes from HSV-1.

As it was the case with the human cellular gene expression, the number of differentially expressed bovine cellular genes increased as infection progressed (Figure 3C, Table S2). It was determined that 10% of the significantly differentially expressed genes overlapped for the three timepoints, while nearly 50% of the total significantly regulated genes were exclusively shared between 6 hpi and 9 hpi (Figure 3D). Transcription factor activation analysis revealed activation of several FoxO family transcription factors; FoxO3a, for instance, has been implicated in the antiviral response [38] during infection as well as NFKB1 during 2 hpi and 6 hpi (Figure 3E, Table S3).

In order to contextualize these results, a pathway analysis was conducted to identify positively or negatively enriched pathways. The Hallmark and Reactome pathways for bovines, which were inferred computationally from homologous human genes, were used for pathway analysis. As illustrated in Figure 3F and further elaborated on in Figure S3, significantly upregulated pathways were already evident at 2 hpi. These pathways included TNF alpha signalling via NFkB, eukaryotic translation initiation and elongation, and MYC targets, among numerous others. A significant negative enrichment of pathways involved in histone deacetylation and methylation and base excision repair, amongst others, was also observed at 2 hpi. As shown in Figure 3F (and Figure S3), at 6 hpi, TNFA signalling via NFkB, translation initiation, and elongation pathways were still upregulated. At 9 hpi, additional upregulation of genes involved in the regulation of expression of SLITs and ROBOs was observed, along with chromatin modifications during the MZT. Infection-related pathways were also positively enriched, including influenza and SARS-CoV-1-linked host translation machinery modulation (see Figure S3, Table S4). Pathway analysis based on ORA was also carried out, showing some overlap between pathways, but also some pathways that were exclusively found for each method (Figure S4A,B, Table S5).

**Figure 4:**
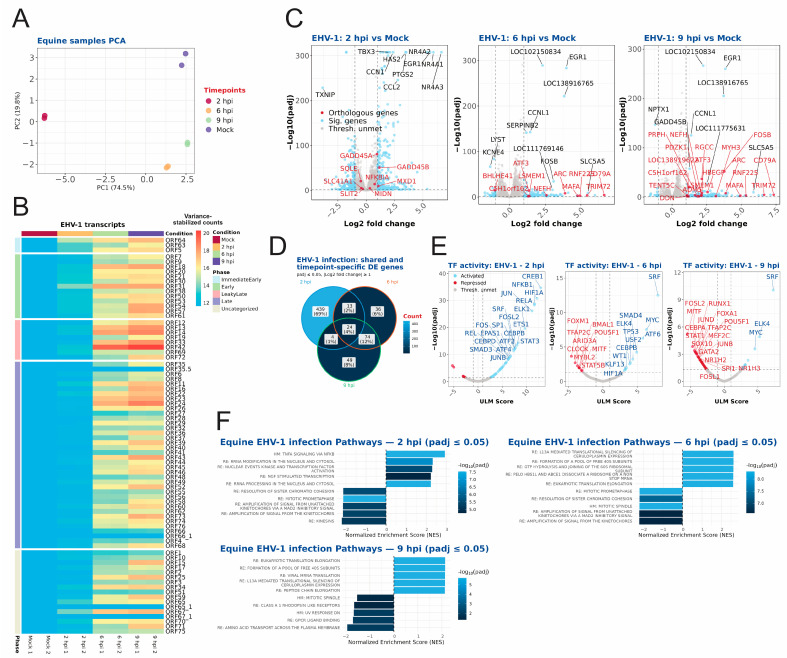
RNA sequencing analysis of equine EDerm cells infected with EHV-1. (**A**) Principal component analysis shown for the different timepoints and replicates of the equine samples, with the variance shown for the principal components. (**B**) EHV-1 transcripts for each timepoint and replicate. The transcripts are ordered according to their respective phase in viral infection; the phase classification is based upon the orthologous genes in human HSV-1 and their respective phase classification. (**C**) Volcano plots shown for the 2 hpi, 6 hpi, and 9 hpi timepoints, with significantly (*p*adj ≤ 0.05) differentially expressed genes (absolute log2 fold change of >1) shown in blue. Genes that have significantly regulated orthologous genes in all three species are depicted in red and genes below the thresholds are shown in grey. (**D**) Venn diagram showing significantly regulated (absolute log2 fold change of ≥1 and *p*adj ≤ 0.05) genes that overlap between the different timepoints of infection. (**E**) Transcription factor activity inference plots for 2 hpi, 6 hpi, and 9 hpi with the Univariate Linear Model (ULM) score plotted with the *p*adj value. Activated transcription factors are shown in blue (ULM score ≥ 1 and *p*adj ≤ 0.05) and repressed transcription factors are shown in red (ULM score ≤ −1 and *p*adj ≤ 0.05). Transcription factors below the thresholds are shown in grey. (**F**) The most enriched significant (*p*adj ≤ 0.05) pathways for each infection timepoint (2 hpi, 6 hpi, and 9 hpi) are shown. For each timepoint, a maximum of 5 positively enriched and a maximum of 5 negatively enriched pathways, each with the largest absolute normalized enrichment score, are shown. All significant pathways are shown in Figure S5.

For EHV-1-infected equine cells, the PCA of the samples demonstrated clear separation between the times post infection, with replicates of each exhibiting close clustering (Figure 4A). As infection progressed, the number of EHV-1 genes expressed increased with the increase in individual transcript abundance for most genes (Figure 4B). Consistent with the lower percentage of EHV-1 reads, EHV-1 genes were generally expressed to lower levels than those of HSV-1 or BHV-1, particularly at 9 hpi (Figure 1C).

In contrast to human or bovine cellular gene expression, the number of differentially expressed equine cellular genes decreased from 2 hpi to 9 hpi (Figure 4C, Table S2). Only 4% of significantly differentially regulated genes were shared among all three timepoints, while each timepoint also had some exclusively regulated genes (Figure 4D). Transcription factor activation analysis revealed activation of MYC, an important proto-oncogene [39], at 6 hpi and 9 hpi, as well as repression of JUNB, which is involved in the immune response and tumorgenesis [40], at 9 hpi (Figure 4E, Table S3).

The Hallmark and Reactome pathways for equines, which were also inferred computationally from homologous human genes, were used for pathway analysis to contextualize the differential gene expression data. As illustrated in Figure 4F and further elaborated on in Figure S5 (Table S4), at 2 hpi there was an increased abundance of transcripts associated with activation of TNFA signalling via NFKB, interleukin 10 signalling, rRNA processing, and MYC-related pathways. This was accompanied by downregulation of transcripts associated with mitotic spindle, M Phase, and cell cycle pathways, among others. At 6 hpi, the translation initiation and elongation pathways of eukaryotes were among the most significantly enriched pathways. In addition, pathways related to nonsense-mediated decay and several infection-related pathways were also positively enriched. At 6 hpi, there was still significant positive enrichment of translation initiation and elongation pathways and infection-related pathways, and downregulation of pathways related to mitosis and the cell cycle. A cursory examination of gene expression at 9 hpi revealed that fewer pathways were subject to differential regulation. Pathways associated with viral infection, those involved in translation initiation and elongation, as well as regulation of expression of SLITs and ROBOs nonetheless remained significantly enriched this time. It has been demonstrated that amino acid transport across the plasma membrane is subject to downregulation. Pathway analysis based on ORA methodology was performed, showing minimal overlap of pathways between gsea and ORA methods (Figure S6A,B, Table S5). Overall, the gsea-based pathway analysis showed more significant pathways for all three timepoints compared to the ORA-based analysis.

**Figure 5:**
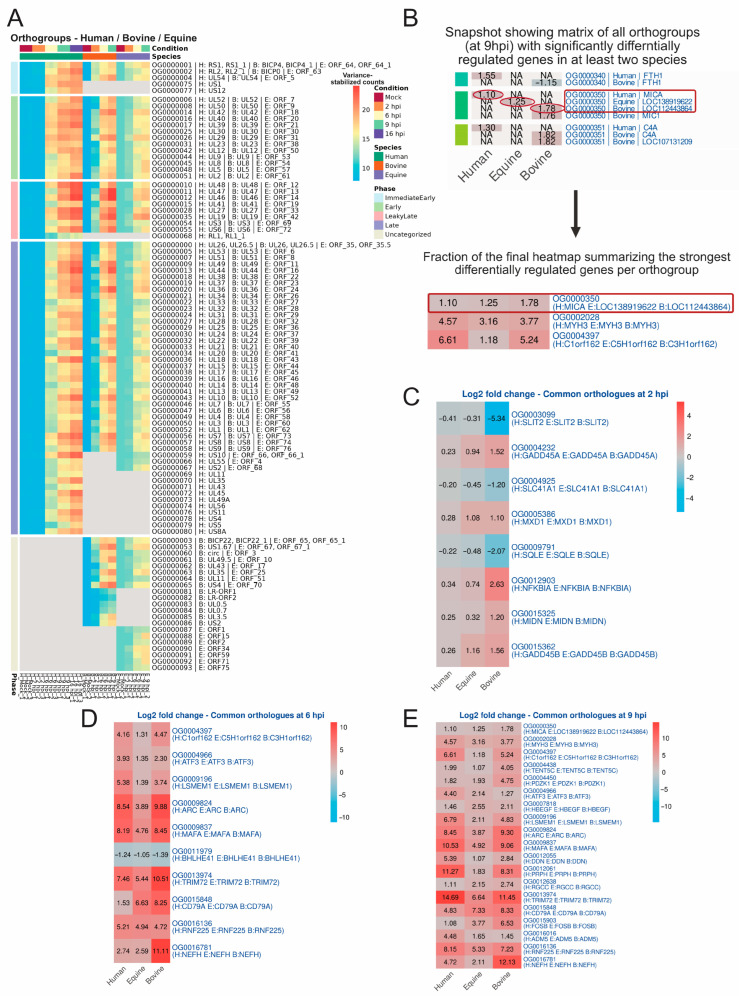
Orthology analysis of viral transcripts, differentially expressed genes, and pathways. (**A**) Orthologous viral transcripts phase classification fully based upon HSV-1 gene classification for comparing different dynamics of orthologous genes to HSV-1. (**B**) Illustration showing a snapshot of the matrix of all orthogroups with significantly differentially regulated genes in at least two species, exemplifying how the genes shown in the final matrix are selected (relevant genes for the final matrix are encircled in red) by keeping the most strongly regulated gene in the orthogroup per species according to the absolute log2 fold change. (**C**) Matrix showing significantly (*p*adj ≤ 0.05) regulated genes in the same orthogroup for human, equine, and bovine samples at 2 hpi. A threshold of an absolute log2 fold change of 0.2 was applied. (**D**) Matrix showing significantly (*p*adj ≤ 0.05) regulated genes that are in the same orthogroup for human, equine, and bovine samples at 6 hpi. A threshold of an absolute log2 fold change of 1 was applied. (**E**) Matrix showing significantly (*p*adj ≤ 0.05) regulated genes that are in the same orthogroup for human, equine, and bovine samples at 9 hpi. A threshold of an absolute log2 fold change of 1 was applied.

Next, we investigated the common pathways altered during alphaherpesvirus infection across these three species. A clustering approach was employed to identify commonly differentially regulated genes within each species’ protein-coding genes as well as the viral genes. For the latter, a shared plot was generated that showed viral genes from the three species, categorized by common orthogroup and segregated into the kinetic class of the corresponding HSV-1 gene (Figure 5A). To identify commonly regulated genes among cellular protein-coding genes, in each result of differential expression, genes with a *p*adj of at most 0.05 and a minimum absolute log2 fold change of 1 were retained. However, in HSV-1-infected HFF-1 cells at 2 hpi, no differentially regulated gene exceeded the absolute log2 fold change threshold of 1 (Figure 2C). Therefore, the log2 fold change threshold was decreased to 0.2 exclusively for the comparison of genes at 2 hpi. A large matrix containing all orthogroups and genes that met these outlined criteria was created for orthogroups that had significant genes in at least two of the three species (Data S1, Table S6). Based on this matrix, the strongest regulated genes per species and per orthogroup were selected, under the condition that all three species must at least have one significantly regulated gene in the respective orthogroup (Figure 5B).

As illustrated in Figure 5C, three genes (SLIT2, SLC41A1, and SQLE) were commonly downregulated, whilst five genes (GADD45A, GADD45B, MXD1, NFKBIA, and MIDN) were commonly upregulated in human, bovine, or equine cells at 2 hpi. At 6 hpi, nine differentially regulated genes were commonly upregulated (C1orf162 (C5H1orf2 in equine samples and C3H1orf162 in bovine samples). These genes were ATF3, LSMEM1, ARC, MAFA, TRIM72, CD79A, RNF225, and NEFH) (Figure 5D). Only one differentially regulated gene (BHLHE41) was commonly downregulated (Figure 5D). Finally, at 9 hpi, 19 differentially regulated genes were commonly upregulated (Figure 5E). These genes included MICA (LOC138919622 in equine samples and LOC112443864 in bovine samples), MYH3, C1orf162 (C5H1orf2 in equine samples and C3H1orf162 in bovine samples), TENT5C, PDZK1, ATF3, HBEGF, LSMEM1, ARC, MAFA, DDN, PRPH, RGCC, TRIM72, CD79A, FOSB, ADM5, RNF225, and NEFH (Figure 5E). No differentially regulated genes were commonly downregulated at this time.

**Figure 6:**
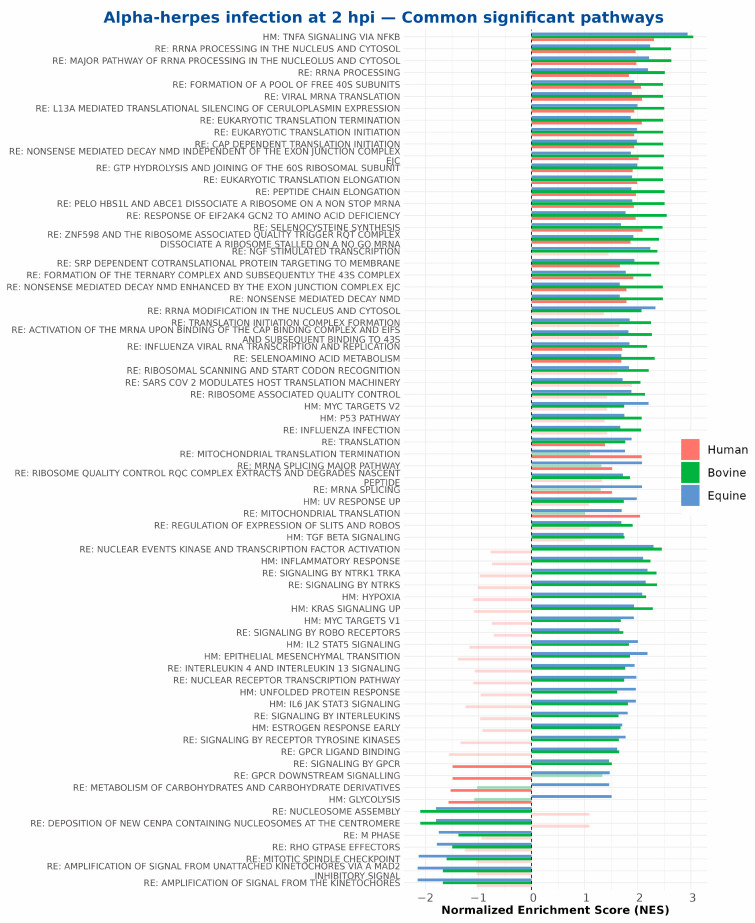
Commonly regulated pathways at 2 hpi for human (orange), bovine (green) and equine (blue) samples where at least two species need to have the same pathway regulated significantly. Bars which are opaque are significant (*p*adj ≤ 0.05), while transparent bars are non-significant.

**Figure 7:**
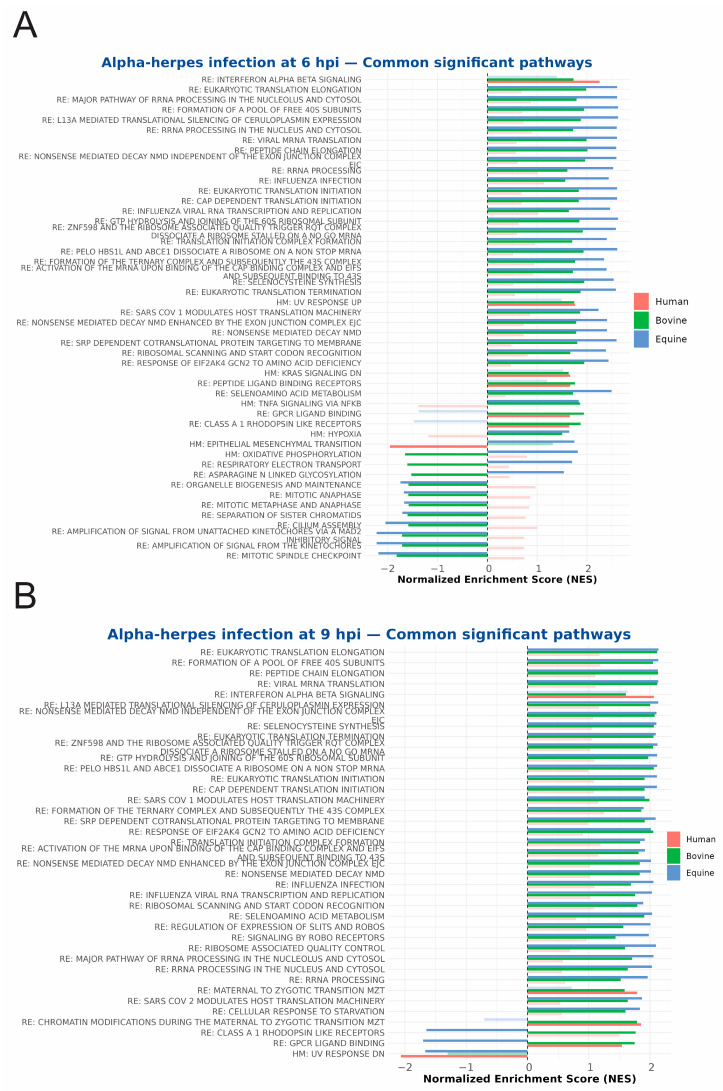
(**A**) Commonly regulated pathways at 6 hpi for human (orange), bovine (green), and equine (blue) samples where at least two species need to have the same pathway regulated significantly. Bars which are opaque are significant (*p*adj ≤ 0.05), while transparent bars are non-significant. (**B**) Commonly regulated pathways at 9 hpi.

Commonly regulated pathways were extracted on the basis of their significant enrichment (*p*adj ≤ 0.05) in a minimum of two of the three species. As illustrated in Figure 6, at 2 hpi, all three species exhibited an increase in the expression of genes involved in TNFA signalling via NFKB, rRNA processing, eukaryotic translation initiation, and response of eIF2 kinase GCN2 to amino acid deficiency pathways. Notably, bovine and equine cells shared more commonly enriched pathways at this time, including MYC targets and the p53 pathway, among others. No pathways were commonly negatively enriched among all three species. At 6 hpi, as illustrated in Figure 7A, no pathways were commonly enriched within human, bovine, or equine cells. TNFA signalling via the NFKB pathway tended to be negatively enriched in human cells, while it remained significantly positively enriched in equine and bovine cells. It is noteworthy that the expression of genes associated with the cell cycle was significantly downregulated at this time in bovine and equine cells. At 9 hpi, both bovine and equine samples showed significantly positively enriched translation initiation and elongation pathways, influenza and SARS-CoV-1/SARS-CoV-2 pathways, and Slits- and Robos-related pathways (Figure 7B). It is also noteworthy that at 9 hpi, bovine and human samples had the GPCR ligand binding pathway significantly positively enriched, whereas in equine samples, this pathway showed significant negative enrichment.

## 3. Discussion

We comprehensively compared the temporal transcriptomes of BHV-1-, EHV-1-, and HSV-1-infected bovine, equine and human cells, respectively. We identified comparable infection levels between HSV-1 and BHV-1, as determined by the percent of viral reads at each evaluated time post infection (Figure 1C). For EHV-1 infection, the percent of viral reads was lower at 6 and 9 hpi, which might be a consequence of the strong host shut-off induced by HSV-1 and BHV-1 vhs proteins. EHV-1 host shut-off is less pronounced than for other alphaherpesviruses as EHV-1 ORF19, the vhs homologue, has lower levels packaged into viral particles [41]. Alternatively, the EHV-1 strain used may be less well-adapted to replication in cell culture than the lab-adapted HSV-1 KOS and BHV-1 Cooper strains. We also observed significant differences in the expression kinetics of viral proteins upon infection. Whereas mostly immediate–early genes (ICP0, ICP4, ICP27 and ICP22) were robustly expressed at 2 hpi with HSV-1, EHV-1-infected cells also expressed early genes, while BHV-1-infected cells had pronounced late gene expression (Figure 2A, Figure 3A, Figure 4A and Figure 5A). Our temporal classification of BHV-1 and EHV-1 was based solely on orthologous HSV-1 genes, since complete annotations into temporal classes are missing for BHV-1 and EHV-1 genomes. Our classification facilitates comparison of gene expression kinetics across viruses, and the results show that the temporal expression of viral genes is not strictly conserved for BHV-1 or EHV-1 orthologous genes. For the latter viruses, temporal expression kinetics are best described for orthologues of the HSV-1 IE proteins ICP4, ICP0, ICP22, and ICP27. Only ICP4 is conserved as an IE protein among all three viruses [42,43]. ICP0 orthologues are expressed as an IE gene for BHV-1, and E genes for BHV-1 and EHV-1 [42,44,45]. ICP27 orthologues in BHV-1 (UL54) and EHV-1 (UL3), meanwhile, are expressed as E proteins [46,47]. ICP22 orthologues are, however, expressed with IE or E gene kinetics in BHV-1 or EHV-1, respectively, and also with L gene kinetics in both viruses [42,48,49,50]. The different kinetics of regulatory protein expression among HSV-1, BHV-1, and EHV-1 provide unique opportunities to comparatively evaluate how these viruses counteract cellular antiviral responses and establish a cellular environment conducive to viral replication.

In order to identify orthologous host genes that are differentially regulated between all three virus infections, we used OrthoFinder. OrthoFinder identifies orthologs by performing an all-vs.-all protein sequence similarity search and clustering genes into orthogroups using graph-based methods. It then constructs multiple sequence alignments and gene trees for each orthogroup and reconciles these with a species tree to distinguish orthologs from paralogs. This species tree-aware framework enables accurate inference of gene evolutionary relationships across multiple genomes. Using OrthoFinder, we identified several orthologous gene groups that are differentially regulated for all viruses.

Two examples of host genes that are transcriptionally induced by all evaluated viruses are ARC (activity-regulated cytoskeleton-associated protein) and ATF3 (activating transcription factor 3, at 6 and 9 hpi). The co-upregulation of these, and other, genes in different cell types (epithelial or fibroblast) derived from evolutionarily distinct species (human, bovine, or equine) infected with alphaherpesviruses of distinct genera (*Simplexvirus* or *Varicellovirus*) supports the idea of evolutionarily conserved cellular responses to alphaherpesvirus infection.

The ARC protein is thought to derive from retrotransposons and plays a key role in synaptic plasticity in neurons by its function of AMPA-type glutamate receptors through the endocytic machinery. A recent study suggested that the protein was shown to form virus-like capsid structures that can bind RNA and shuttle it across the endosomal membrane [51]. The upregulation of ARC expression during HSV-1 infection has previously been reported and is induced by ICP0, an HSV-1 IE protein [52]. Knockdown of ARC negatively affects HSV-1 replication capacity at early infection stages [52]. BHV-1 and EHV-1 encode ICP0 homologues that share 18% or 13% amino acid sequence identity with HSV-1 ICP0, respectively. BICP0 is expressed with IE and E kinetics, whereas eICP0 is expressed with E kinetics [42,44]. As BHV-1 and EHV-1 also upregulate ARC, it would be interesting to mechanistically characterize how ARC is upregulated and comparatively evaluate its roles during veterinary alphaherpesvirus infection.

ATF3 is an immediate–early response gene that is upregulated in minutes in response to various stimuli. ATF3 is involved in the stress response of multiple different cell types [53]. Its expression is activated consequent to a wide array of stimuli, including proteasome inhibitors [54], nerve damage [55], and amino acid starvation [56]. ATF3 also plays roles in nerve regeneration [57]. Since HSV-1, BHV-1, and EHV-1 are neurotropic viruses that establish latency in neurons, it is of great interest that all viruses induce expression of ATF3 that has a neuro-protective role. This points towards a common mechanism in which alphaherpesviruses may induce survival of infected neurons to support maintenance of latent infection. Consistently, Shu et al. showed that ATF3 is involved in inducing HSV-1 LAT gene expression to maintain latency and suggested that ATF3 promotes neuronal integrity upon cellular stress to avoid neuronal death [58].

For pathway analysis, we used the Reactome and Hallway pathway datasets. Reactome pathway analysis offers mechanistic, expert-curated pathways that show how genes interact in ordered biological reactions, whereas more simple analyses like GeneOntology (GO) mainly provide hierarchical annotations without pathway structure. Because Reactome models pathway topology, they can assess functional impact with greater precision. Reactome pathways tend to be less redundant and more cohesive, making results easier to interpret. This type of analysis also includes disease pathways, drug interactions, and high-quality visualizations that GO generally lacks. With our analysis, we identified several pathways that are jointly regulated by all three virus infections, especially at the earliest sampled timepoint of infection, 2 hpi. Here, we could find pathways related to translation rRNA processing and TNF-alpha signalling that were enriched for all three virus infections. In the 6 hpi timepoint, bovine and equine infections share a larger proportion of commonly enriched pathways, which are also related to rRNA processing, translation, and viral infections. There were no significant pathways enriched in the same direction shared between equine and human infections, while there were some enriched pathways like interferon alpha and beta signalling which were shared between bovine and human infections. At 9 hpi, a lot of the same pathways that are also enriched at 6 hpi are still shared between bovine and equine samples. Exemplary pathways that may open up new avenues for antiviral therapies are the “Cellular response to starvation” significantly upregulated in bovine and equine samples at 9 hpi as well as the “maternal-to-zygotic transition” (MZT) shared between bovine and human samples at 9 hpi. The starvation pathway may be of interest, as parts of the autophagy machinery have been shown to be hijacked by viruses supporting viral replication [59,60]. It was demonstrated that HSV-1 actively modulates the autophagic machinery to enhance its replication and evade innate immune responses, so restoring or manipulating autophagic flux may limit viral propagation. Pharmacological agents such as rapamycin (autophagy induction via mTOR inhibition), chloroquine or hydroxychloroquine (blocking autophagosome–lysosome fusion), and spermidine (autophagy activation) have therefore been proposed as potential adjunct antiviral strategies against HSV-1 and could also be tested for animal BHV-1 and EHV-1. Moreover, the enrichment of MTZ pathways hints at a possible common viral adaptation to induce maternal-to-zygotic transition genes (Figure 7B). Putting this into context, we have recently shown for human herpesviruses that viral mimicry of MZT through viral activation of DUX4 is crucial for HSV-1 replication, and that DUX4 could be a target for HSV-1 therapy [61]. Further studies are needed to evaluate the functional role of individual pathways in animal herpesvirus infections and whether therapeutic intervention could be an option for antiviral therapy in the future.

## 4. Materials and Methods

### 4.1. Viruses and Cells

EHV-1 strain R08-8428 and BHV-1 strain Cooper were generous gifts from Dr. Vikram Misra (University of Saskatchewan). Primary HFF-1 (SCRC-1041) was acquired from ATCC and was maintained in Dulbecco’s modified Eagle’s medium (DMEM, Thermo Fisher Scientific, Darmstadt, Germany) supplemented with 10% (vol/vol) heat-inactivated fetal bovine serum (FBS, Thermo Fisher Scientific), 2 mM GlutaMAX (Thermo Fisher Scientific), 1 mM HEPES (Thermo Fisher Scientific) and 1% (vol/vol) gentamycin or penicillin–streptomycin (vol/vol). HSV-1 strain KOS was used for infection of HFF-1 cells.

EHV-1 strain R08-8428 has ORF 30 A2254 (N752) associated with non-neurotropic EHV-1 pathotype [62,63]. Equine dermal (EDerm) cells (NBL-6; ATCC) were maintained in Dulbecco’s modified minimum Eagle’s medium (DMEM, Gibco, Carlsbad, CA, USA 11885-084) supplemented with 10% fetal bovine serum (FBS, Corning, Glendale, AZ, USA 35-077-CV) at 37 °C in 5% CO_2_. Bovine kidney epithelial (MDBK) cells (NBL-1; ATCC) were maintained in DMEM supplemented with 10% heat-inactivated horse serum (HS, Gibco 26050-088) at 37 °C in 5% CO_2_. MDBK cells are bovine viral diarrhea virus (BVDV)-free.

### 4.2. Viral Stock Preparation and Titration

EHV-1 or BHV-1 stocks were prepared and titrated in EDerm or MDBK cells, respectively. Briefly, cells were infected with 0.01 to 0.05 plaque-forming units (PFUs) per cell diluted in 4 °C DMEM. Cells were incubated with inoculum, and underwent rocking and rotating every 5–10 min, for 1 h at 37 °C in 5% CO_2_. Following inoculum removal, cells were washed with 4 °C phosphate-buffered saline (PBS; 150 mM NaCl, 1 mM KH_2_PO_4_, 3 mM Na_2_HPO_4_, pH 7.4) two times, fresh 37 °C DMEM supplemented with 10% FBS (EHV1) or 10% HS (BHV-1) was added, and then cells were incubated at 33 °C in 5% CO_2_. When a cytopathic effect of over 95% was observed, cells were harvested and pelleted by centrifugation at 3214× *g* for 30 min at 4 °C. The resulting supernatant was centrifuged at 10,000× *g* for 2 h at 4 °C to pellet extracellular virions. The cell pellet was disrupted through three rapid freeze–thaw cycles to release intracellular virions. Virions were isolated from cellular debris by centrifugation at 5500× *g* for 30 min at 4 °C. Extra- and intracellular virions were combined for viral stocks. Viral stocks were titrated as described [64].

### 4.3. Infection Experiments

EDerm or MDBK cells were seeded in 100 mm dishes such that they would be approximately 80% confluent at the time of infection. Purified EHV-1 or BHV-1 stocks were diluted to 10 plaque-forming units per cell (PFUs/cell) in 4 °C DMEM. Cells were overlayed with inoculum, or 4 °C DMEM for mock infection, incubated at 37 °C in 5% CO_2_ for 1 h with rocking and rotating every 5 to 10 min, then inoculum was removed and cells were washed twice with 4 °C PBS. Cells were overlayed with fresh 37 °C DMEM supplemented with 10% FBS (EDerm) or 10% HS (MDBK) and incubated at 37 °C in 5% CO_2_ until harvest. Both EDerm and MDBK mock-treated cells were harvested at the 9 h post-infection timepoint. HFF-1 cells were seeded in 6-well plates with 0.330 × 10^6^ cells/well. HSV-1 KOS stocks were diluted to 3 PFUs/cell and HFF-1 cells either infected or mock-treated (harvested 6 h post mock treatment) and then incubated at 37 °C in 5% CO_2_.

### 4.4. Cell Harvest, RNA Isolation, and Library Preparation

Cells were washed in 4 °C PBS then scraped and collected in TRIzol Reagent (Invitrogen, Carlsbad, CA, USA 15596026). Samples were frozen and stored at −80 °C until samples from all times were collected. Prior to RNA isolation and purification, samples were thawed and incubated at room temperature for at least 5 min. RNA was isolated and purified according to the manufacturers’ instructions (RNA clean and concentrator kit Zymo Research). Oligo(dT) beads were used to bind to the poly(A) tails and enrich mature mRNAs and lncRNAs. Sequencing libraries were prepared using the NEBNext Ultra II Directional RNA Library Prep Kit for Illumina (NEB, Frankfurt am Main, Germany) with 9 cycles of PCR amplification and sequenced on an Illumina Novaseq X or a DNBSEQ-T7 sequencer, both with 2 × 50 cycles for a read depth of 30 M. Both EDerm and MDBK samples are technical replicates at the library-prep level, while HFF-1 samples are biological replicates. RNA sequencing samples was sent to Biomarkers Technologies (BMKGENE, Münster, Germany) for sequencing.

### 4.5. Transcriptomics Data Preparation

For the processing of the total RNAs sequencing data, the mRNAseq pipeline of snakePipes (version 3.1.0) was employed [65]. The snakePipes pipeline performed adapter and base quality trimming through TrimGalore (0.6.10) [66] and the trimmed reads were aligned through the STAR (2.7.10b) aligner [67] to a hybrid genome. For human samples, the hybrid genome consisted of the human hg38 genome (GRCh38.p14, Release 47) and HSV-1 KOS genome (GenBank: JQ673480.1, assembly: GCA_027937515.1), the latter having the terminal repeat regions masked (1–9161 and 145,394–152,010). For bovine, the ARS-UCD2.0 (GCF_002263795.3) genome was used merged with the BHV-1 genome (GenBank: JX898220.1, assembly: GCA_008777455.1) which had its terminal repeat region masked (123,906–134,896). For equine samples, we used the TB-T2T genome (GCF_041296265.1) with the EHV-1 genome (GenBank: AY665713.1; assembly: GCA_000844025.1) with the terminal repeats masked (1–31 and 137,511–150,224). For all viral annotation .gtf files, if no exons were annotated, the “CDS” attributes were changed to “exon”, for later feature counting by featureCounts (v2.0.1) [68]. The featureCounts tool was used to generate the count matrix for analysis with the following options: “-C -Q 0 --primary -M”. Bigwig files were generated through deepTools’ (3.5.4) bamCoverage [69] with scale factors calculated by the DESeq2 package [70]. For the orthogroups, OrthoFinder (v3.1.0) [34,35] was employed with standard settings, with each species’ primary transcript as input. The primary transcript files were generated from the FASTA files with annotated CDS features translated to proteins from NCBI’s Assembly resource (https://ftp.ncbi.nlm.nih.gov/genomes/ (accessed on 30 October 2025): GCF_000001405.40_Human_GRCh38.p14_translated_cds.faa, GCF_002263795.3_Bovine_ARS-UCD2.0_translated_cds.faa and GCF_041296265.1_Horse_TB-T2T_translated_cds.faa) with the primary_transcript.py script included in OrthoFinder. For the orthogroups of the three viruses, the same procedure was performed with fasta files containing the virus’ protein sequences. General statistics of the data preparation process were generated with miltiqc v1.23. RNA integrity was checked via gene body coverage graphs for each species, making sure that read coverage over gene bodies is similar, ruling out RNA degradation as a source of differences in gene expression over the infectious time course. For this, geneBody_coverage2.py from RSeQC (v5.0.4) [71] was used with the bam files generated by bamCoverage without any normalization applied.

### 4.6. Transcriptomics Data Analysis

The counts table was filtered, retaining features with five or more counts in at least two data points. Data filtering and processing were mainly performed via the dplyr (1.1.4) R library [72] in RStudio (2024.12.0 Build 467) [72,73] with R version 4.5.2 [74]. DESeq2 (1.48.2) [70] R package was used for the principal component analysis (PCA), viral transcript plots, and calculating fold changes, as well as their respective significance. DESeq2 scaling factors were calculated based on host factors removing any effect viral genes may have. As a design formula, only the timepoint of infection was used. For the viral transcript plots, variance-stabilizing transformation (VST), contained in the DESeq2 (1.48.2), was used to transform the counts and plotted with the pheatmap (1.0.13) R library [75]. The top 500 most variable features, based on VST counts, were used as input for the PCA. For the human genes, the Ensemble Gene IDs were converted to the external gene names through the biomaRt (2.64.0) package [76]. For each calculation of the foldchange, the lfcShrinkage function with the apeglm algorithm was used with the appropriate comparison (timepoint vs. mock) set as the coef. Also, *p*adj values of 0 were set to Machine$double.xmin and viral transcripts were removed for the visualization, which was performed with ggplot2 (4.0.1) [77].

### 4.7. Pathway Analysis

Pathway analysis was performed based on Hallmark and Reactome pathways. To obtain the human hallmark pathways, the R library MSigDB [78] (version 25.1.1) was used, while for the Reactome data, a custom script was used in order to obtain the most recent Reactome (v94) data [79]. For the Reactome data, only human pathways with their respective genes were kept, while for the 1:1 mapping of human genes to other species, the most recent HCOP (last updated on the 26 July 2024, from https://ftp.ebi.ac.uk/pub/databases/genenames/hcop/human_all_hcop_sixteen_column.txt.gz (accessed on the 16 January 2026)) was downloaded [80]. The HCOP dataset was processed similarly to the babelgene R library [81] (which contains the HCOP dataset from 2022) and is used in MSigDB to perform orthology mappings. For the processing of the HCOP dataset, human and species’ symbols and Ensembl gene IDs, as well as the tools supporting the mapping, were extracted (removing duplicates of the entries as well as duplicates among the tools supporting the mapping in each orthologous gene pair). Next, duplicate entries mapping the same genes (based on symbols and Ensembl gene IDs) but containing different tools to support the mapping were merged and entries which had only one single tool supporting the mapping were removed. Next, for human genes with multiple mappings, the entry with the highest number of tools supporting the mapping was retained, while in the case of a tie, all mappings were kept. Finally, entries with no species’ symbol were removed. Based on this orthologous mapping matrix, the human Hallmark and Reactome pathways were converted to the respective species’ pathways. Based on these pathways, fgseaMultilevel (minsize of 15 and maxsize of 500, eps of 1 × 10^−10^, score type “std,” and nPermSimple set to 50,000) from the R library fgsea (1.34.2) [82] was used for gene set enrichment with ranks based on the shrunken log2 fold change from the differential expression results for each infection timepoint. For the human samples, the 16 hpi timepoint was included during filtering (as described above), as well as during shrinking of the log2 fold changes. Common pathways were shown if the same pathway of at least two species was significantly regulated (*p*adj ≤ 0.05) in any enriched direction. For the over-representation analysis, we used the same pathways as before. The differentially expressed gene tables were filtered with a *p*adj threshold of 0.05 and an absolute log2 fold change of at least 1 (with the exception of 2 hpi for the human samples, where the log2 fold change threshold was set to 0.2 because there were no significant genes with a high enough absolute Log2 fold change). The clusterProfiler (4.16.0) enricher function [83] was used to perform the over-representation analysis (with the pAdjustMethod = BH, minGSize = 15, maxGSize = 500, pvalueCutoff = 1 and qvalueCutoff = 1) for up- and downregulated genes separately.

### 4.8. Orthologous Gene Mapping

For the orthologous differentially regulated gene tables, all significantly regulated genes (*p*adj of 0.05) and an absolute log2 fold change of either 0.2 at 2 hpi or 1 for 6 hpi and 9 hpi were used. For each significantly regulated gene, the orthogroup (based on the OrthoFinder v3.1 output) of that gene was used to check whether there are significantly regulated genes for the other species. If that was the case, the genes with the highest log2 fold change (while keeping the majority sign of the differential regulation uniform across species) per orthogroup for each species were taken to generate the plot. Additionally, plots with genes significantly regulated in at least two species were also shown, with all significant genes shown for that orthogroup.

### 4.9. Transcription Factor Activity Analysis

Transcription factor analysis was carried out using the decoupleR (2.14.0) library in R using the Dorothea database, which consisted of signed transcription factors used to target gene interactions [36,37]. The Dorothea levels of confidence A, B, and C were used. For the animals, the orthologous mapping matrix that we described before was used to map animal genes to human genes in order to be able to use the Dorothea database. When there was one animal gene that mapped to several human genes, the differential expression data was duplicated for each human counterpart. If several animal genes mapped to one human gene, the highest abs stat value (as calculated by the results function in DESeq2 before shrinking the log2 fold changes) was used. The stat values of the differential expressions were used to create a matrix serving as an input for the Univariate Linear Model function of decoupleR (run_ulm()). The resulting *p*-values were also adjusted for multiple comparisons through the Benjamini–Hochberg [84] method as implemented in the p.adjust() function in the R stats library.

## Data Availability

The sequencing data (RNA-seq.) generated in this study have been deposited in the GEO database under accession code GSE314009. The source code and shell commands were deposited to the following GitHub: https://github.com/bleblebles/Cross-species-analysis-of-transcriptomic-response-to-alpha-herpesvirus-infection/ (accessed on 16 January 2026).

## Supplementary Materials

The following supporting information can be downloaded at https://www.mdpi.com/article/10.3390/ijms27031261/s1.
