# Supplementary material for: Cross-Species Analysis of Transcriptomic Response to Alpha-Herpesvirus Infection in Human, Bovine and Equine Cells
Source: Int J Mol Sci. 2026 Jan 27;27(3):1261. doi: 10.3390/ijms27031261 (PMC12898550; doi:10.3390/ijms27031261)
Supplement: Supplementary file 1 [file ijms-27-01261-s001.zip › Supp_Figures S1-S6 legends.pdf]

Supp Figure S1. All significantly regulated pathways (gsea method) for human samples at 2hpi, 6hpi and 9hpi having their normalized enrichment score plotted as well as the respective bars' color assigned based on the adjusted p-values (padj).

Supp Figure S2: A. Venn diagram showing the number of significant (padj ≤ 0.05) regulated pathways in either with the gsea methodology (blue) or ORA (separated into up and down regulated, respectively orange and violet). **B.** Pathways as extracted from the ORA-based pathway analysis showing the three timepoints 2hpi, 6hpi and 9hpi from left to right. The upregulated pathways are shown on top and the downregulated pathways at the bottom.

Supp Figure S3. All significantly regulated pathways for (gsea method) bovine samples at 2hpi, 6hpi and 9hpi having their normalized enrichment score plotted as well as the respective bars' color assigned based on the adjusted p-values (padj).

Supp Figure S4: A. Venn diagram showing the number of significant (padj ≤ 0.05) regulated pathways in either with the gsea methodology (blue) or ORA (separated into up and down regulated, respectively orange and violet). **B.** Pathways as extracted from the ORA-based pathway analysis showing the three timepoints 2hpi, 6hpi and 9hpi from left to right. The upregulated pathways are shown on top and the downregulated pathways at the bottom.

Supp Figure S5. All significantly regulated pathways for (gsea method) equine samples at 2hpi, 6hpi and 9hpi having their normalized enrichment score plotted as well as the respective bars' color assigned based on the adjusted p-values (padj).

Supp Figure S6: A. Venn diagram showing the number of significant (padj ≤ 0.05) regulated pathways in either with the gsea methodology (blue) or ORA (separated into up and down regulated, respectively orange and violet). **B.** Pathways as extracted from the ORA-based pathway analysis showing the three timepoints 2hpi, 6hpi and 9hpi from left to right. The upregulated pathways are shown on top and the downregulated pathways at the bottom.
