# Supplementary figures and images for: Cross-Species Analysis of Transcriptomic Response to Alpha-Herpesvirus Infection in Human, Bovine and Equine Cells
Source: Int J Mol Sci. 2026 Jan 27;27(3):1261. doi: 10.3390/ijms27031261 (PMC12898550; doi:10.3390/ijms27031261)

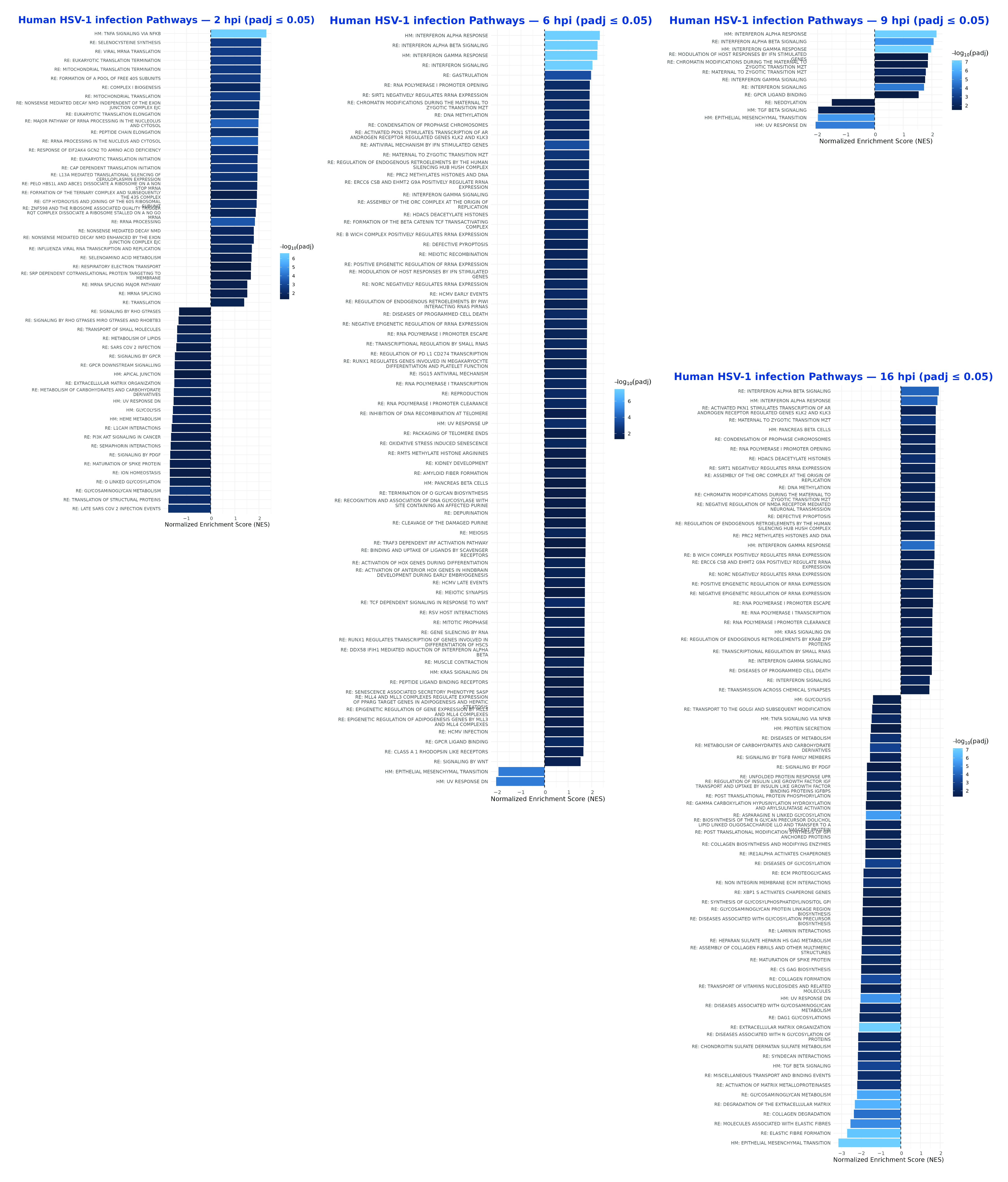

Supplement: Supplementary file 1 [file ijms-27-01261-s001.zip › Supp_Figure S1_human.jpg]

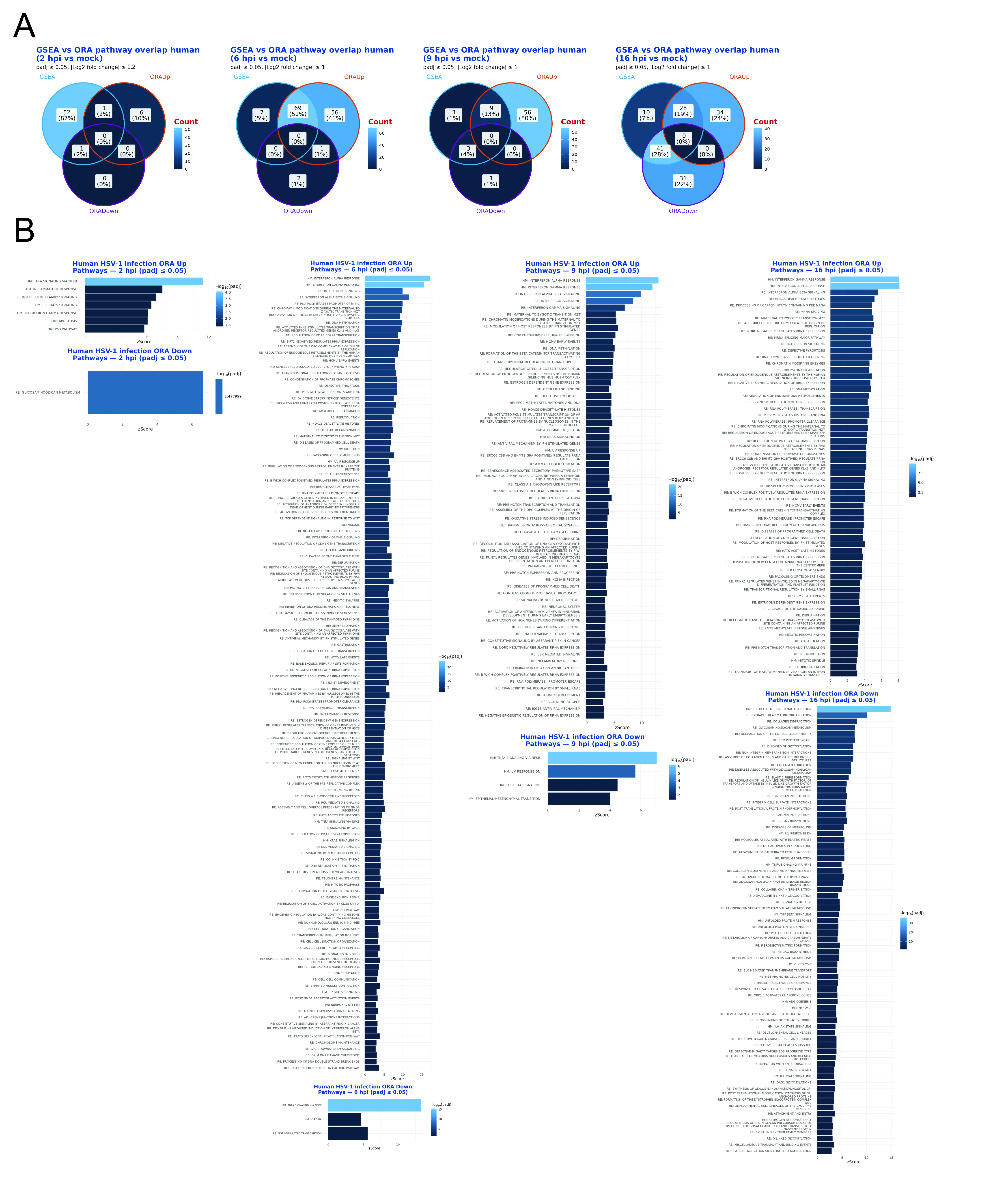

Supplement: Supplementary file 1 [file ijms-27-01261-s001.zip › Supp_Figure S2_human_ORA.jpg]

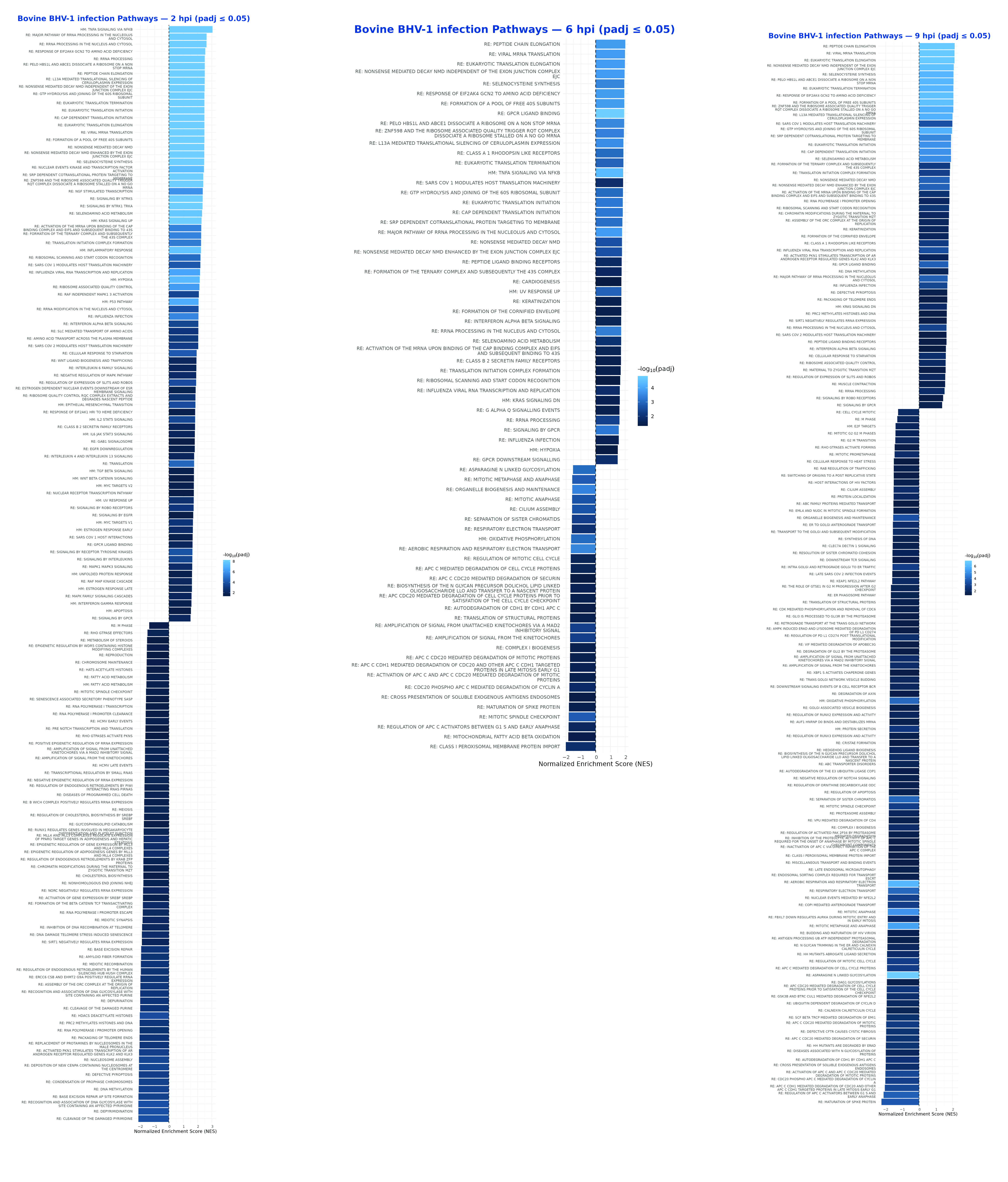

Supplement: Supplementary file 1 [file ijms-27-01261-s001.zip › Supp_Figure S3_bovine.jpg]

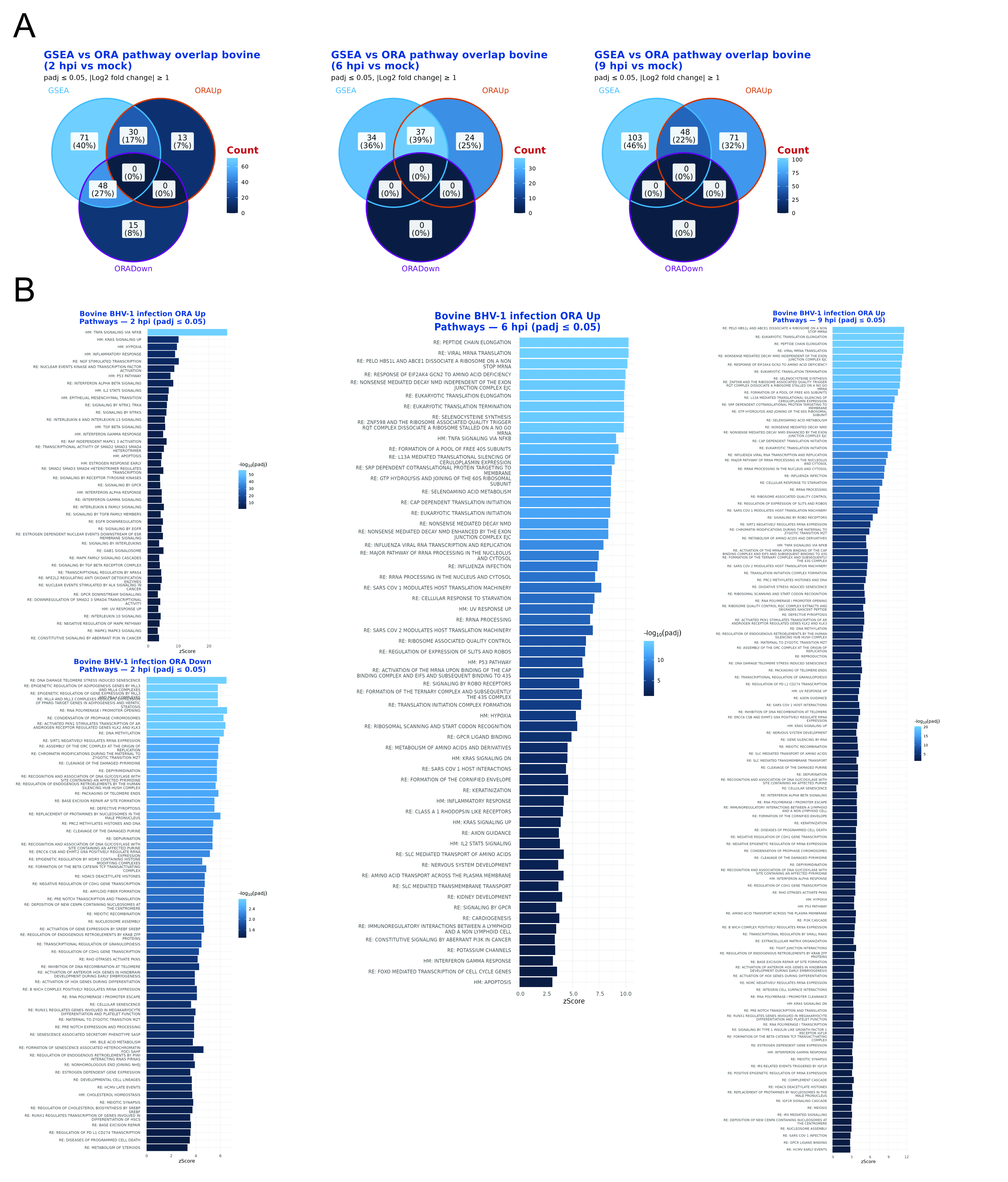

Supplement: Supplementary file 1 [file ijms-27-01261-s001.zip › Supp_Figure S4_bovine_ORA.jpg]

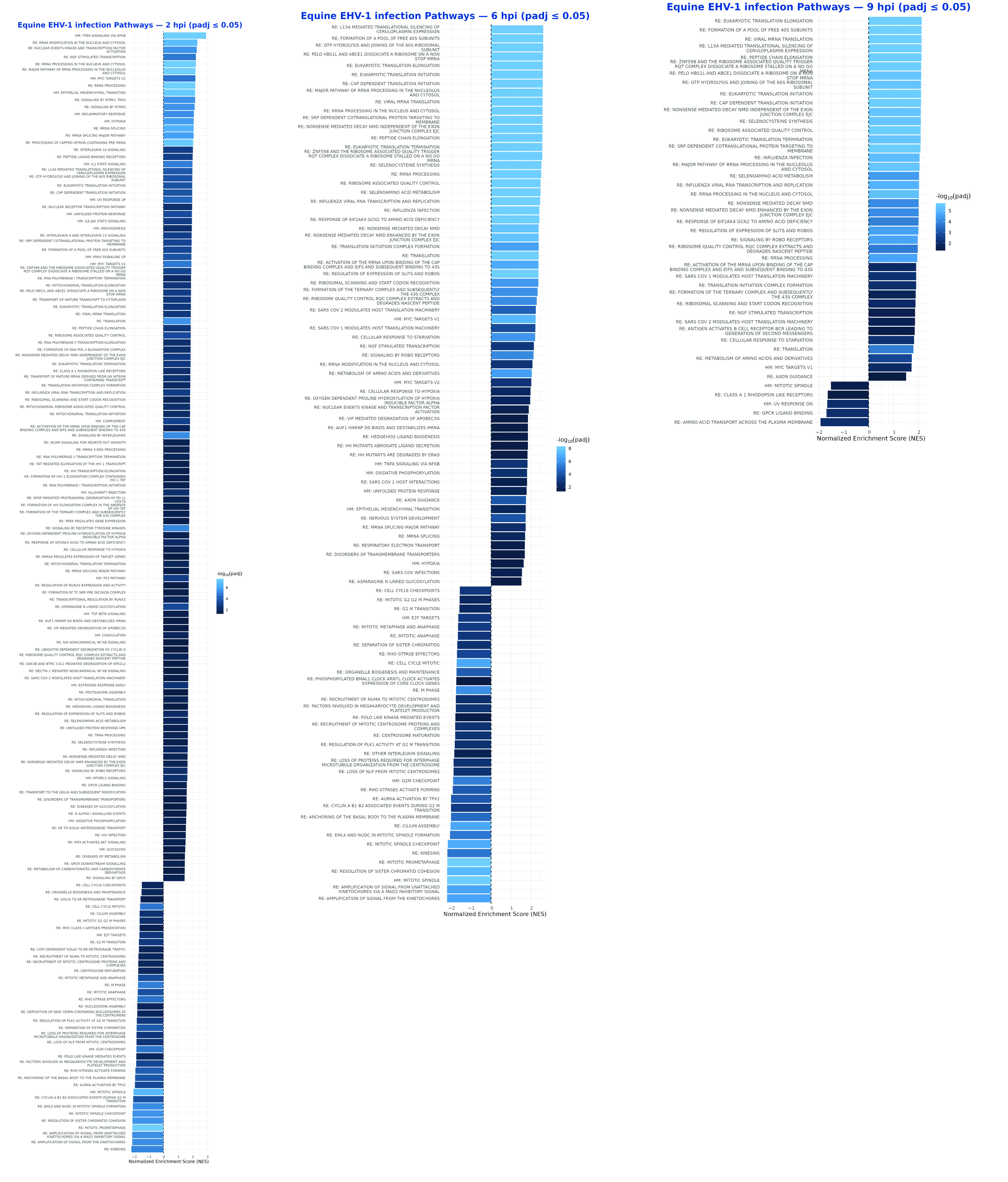

Supplement: Supplementary file 1 [file ijms-27-01261-s001.zip › Supp_Figure S5_equine.jpg]

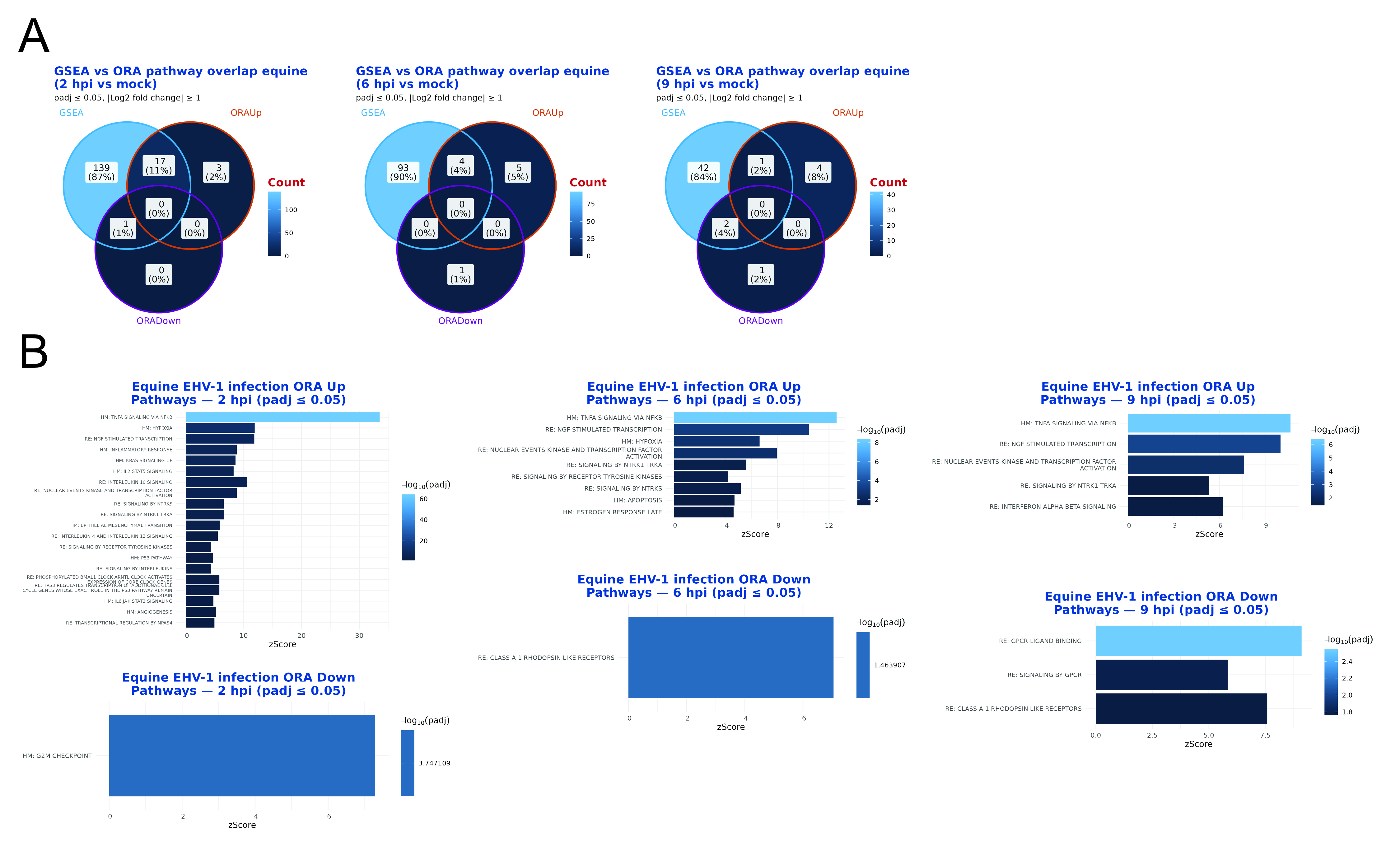

Supplement: Supplementary file 1 [file ijms-27-01261-s001.zip › Supp_Figure S6_equine_ORA.jpg]
